# Unraveling the diversity of sedimentary sulfate-reducing prokaryotes (SRP) across Tibetan saline lakes using epicPCR

**DOI:** 10.1186/s40168-019-0688-4

**Published:** 2019-05-04

**Authors:** Huayu Qin, Shang Wang, Kai Feng, Zhili He, Marko P. J. Virta, Weiguo Hou, Hailiang Dong, Ye Deng

**Affiliations:** 1 CAS Key Laboratory of Environmental Biotechnology, Research Center for Eco-Environmental Sciences, Chinese Academy of Sciences, 18 Shuangqing Rd, Haidian, Beijing, 100085 China; 2 Institute for Marine Science and Technology, Shandong University, Qingdao, 266237 China; 3 College of Resources and Environment, University of Chinese Academy of Sciences, Beijing, 100049 China; 4 School of Environmental Science and Engineering, Sun Yat-Sen University, Guangzhou, 510006 China; 5 Department of Environmental Sciences, University of Helsinki, 00014 Helsinki, Finland; 6 State Key Laboratory of Biogeology and Environmental Geology, China University of Geosciences, Beijing, China; 7 Department of Geology and Environmental Earth Science, Miami University, Oxford, OH United States

**Keywords:** Sulfate-reducing prokaryotes, Functional taxa diversity, epicPCR, Tibetan saline lakes

## Abstract

Sulfate reduction is an important biogeochemical process in the ecosphere; however, the major taxa of sulfate reducers have not been fully identified. Here, we used epicPCR (Emulsion, Paired Isolation, and Concatenation PCR) technology to identify the phylogeny of sulfate-reducing prokaryotes (SRP) in sediments from Tibetan Plateau saline lakes. A total of 12,519 OTUs and 883 SRP-OTUs were detected in ten lakes by sequencing of 16S rRNA gene PCR amplicons and epicPCR products of fused 16S rRNA plus *dsrB* gene, respectively, with *Proteobacteria*, *Firmicutes,* and *Bacteroidetes* being the dominant phyla in both datasets. The 120 highly abundant SRP-OTUs (> 1% in at least one sample) were affiliated with 17 described phyla, only 7 of which are widely recognized as SRP phyla. The majority of OTUs from both the whole microbial communities and the SRPs were not detected in more than one specific lake, suggesting high levels of endemism. The α-diversity of the entire microbial community and SRP sub-community showed significant positive correlations. The pH value and mean water temperature of the month prior to sampling were the environmental determinants for the whole microbial community, while the mean water temperature and total nitrogen were the major environmental drivers for the SRP sub-community. This study revealed there are still many undocumented SRP in Tibetan saline lakes, many of which could be endemic and adapted to specific environmental conditions.

**Electronic supplementary material:**

The online version of this article (10.1186/s40168-019-0688-4) contains supplementary material, which is available to authorized users.

## Introduction

The sulfur cycle is one of the key biogeochemical processes in the ecosphere and has helped to shape the Earth’s geological and biological history. Dissimilatory sulfate reduction, the primary bioprocess within the sulfur cycle, is driven by sulfate-reducing prokaryotes (SRP), which are a diverse taxa group of anaerobic sulfidogenic microorganisms using oxidized sulfur compounds as terminal electron acceptors [1, 2]. They have been intensively studied, especially in marine and freshwater ecosystems for their important applications in bioremediation [3].

Saline lakes at high altitudes are often inhabited by a diverse community of extremophiles involved in various biogeochemical cycles [4–7]. The sulfur cycle, especially sulfur reduction, is very active in saline aquatic environments [7, 8], and many sulfate-reducing bacterial strains have been isolated from the water and sediments of saline lakes [9, 10]. The Tibetan Plateau contains thousands of saline/hypersaline lakes characterized by low nutrients, low temperature, and high UV exposure, providing unique niches for the inhabitation of microorganisms [11]. The microbial communities of Tibetan saline lakes and their relationship with environmental factors have been studied in several lakes [12–15], including the distribution of *Proteobacteria* and *Firmicutes* phyla along vertical depth profiles in sediments of both Qinghai and Chaka lakes [12, 13]. The most influential environmental factors for shaping microbial communities have been observed to be either pH [14] or salinity [15]. A previous study [16] showed that the dissimilatory reduction of oxidized sulfur compounds (sulfate, thiosulfate, and sulfite) and sulfur in saline lakes were mediated by lithotrophic SRP present within the lake sediments. However, the taxonomic identity of SRP in Tibetan saline lake sediments is less clear, and the effects of comprehensive environmental factors on SRP remain unknown.

Although environmental SRP communities have previously been studied using the dissimilatory sulfite reductase gene (*dsrAB*) as a molecular marker [2, 17], a recent study using publicly available metagenomics data has suggested that the distribution of SRP phyla has been significantly underestimated in various ecosystems [18]. To accurately identify SRPs and track their phylogenetic distribution within their respective microbial communities remains a challenge with currently wide-applied techniques. The recently developed epicPCR (Emulsion, Paired Isolation, and Concatenation PCR) technology, which has previously been used to target the SRP sub-community at the single-cell level [19], could be a potential solution. This technique sequesters microorganisms into millions of emulsified droplets, each containing a single microbial cell, amplifies functional (*dsrB*) and phylogenetic (16S rRNA) genes, and fuses the amplified products together within each droplet. Thereafter, all fused PCR products in the droplets are pooled together and sequenced via high-throughput sequencing. Each read contains 16S and functional genes simultaneously, which could allow phylogenetic identification of the functional taxa. It is much more cost-effective for functional taxa discovery in environmental samples than shotgun metagenomic sequencing. The sensitivity of this technique was originally tested by comparing barcode-fused 16S rRNA gene diversity and bulk 16S rRNA sequencing that is able to cover all major phylogenetic groups [19], indicating that the specificity of this technology is fairly high. It has been successfully used to amplify a fusion product of *dsrB* and 16S rRNA genes at the single cell level [19], as well as other functional genes such as those related to antibiotic resistance [20, 21]. By using this approach, the taxonomy and abundance of SRP individuals could be determined simultaneously [19].

In this study, high-throughput sequencing of *dsrB*-16S rRNA gene fusion amplicons by epicPCR and 16S rRNA gene amplicons was performed to explore and compare the diversity of the SRP sub-community and the entire microbial community in surface sediments collected from ten saline lakes of the Tibetan Plateau. These are permanent lakes located distantly from each other (> 391.68 km) and lack direct anthropogenic influences. The aims of the study were to (i) explore the community structure and diversity of SRP groups in Tibetan saline lake sediments, (ii) expand the existing taxonomic breadth of SRP using epicPCR approach, and (iii) evaluate the deterministic environmental factors that shape both the entire microbial communities and SRP sub-communities in Tibetan lakes.

## Materials and methods

### Sampling sites and collection

**Fig. 1:**
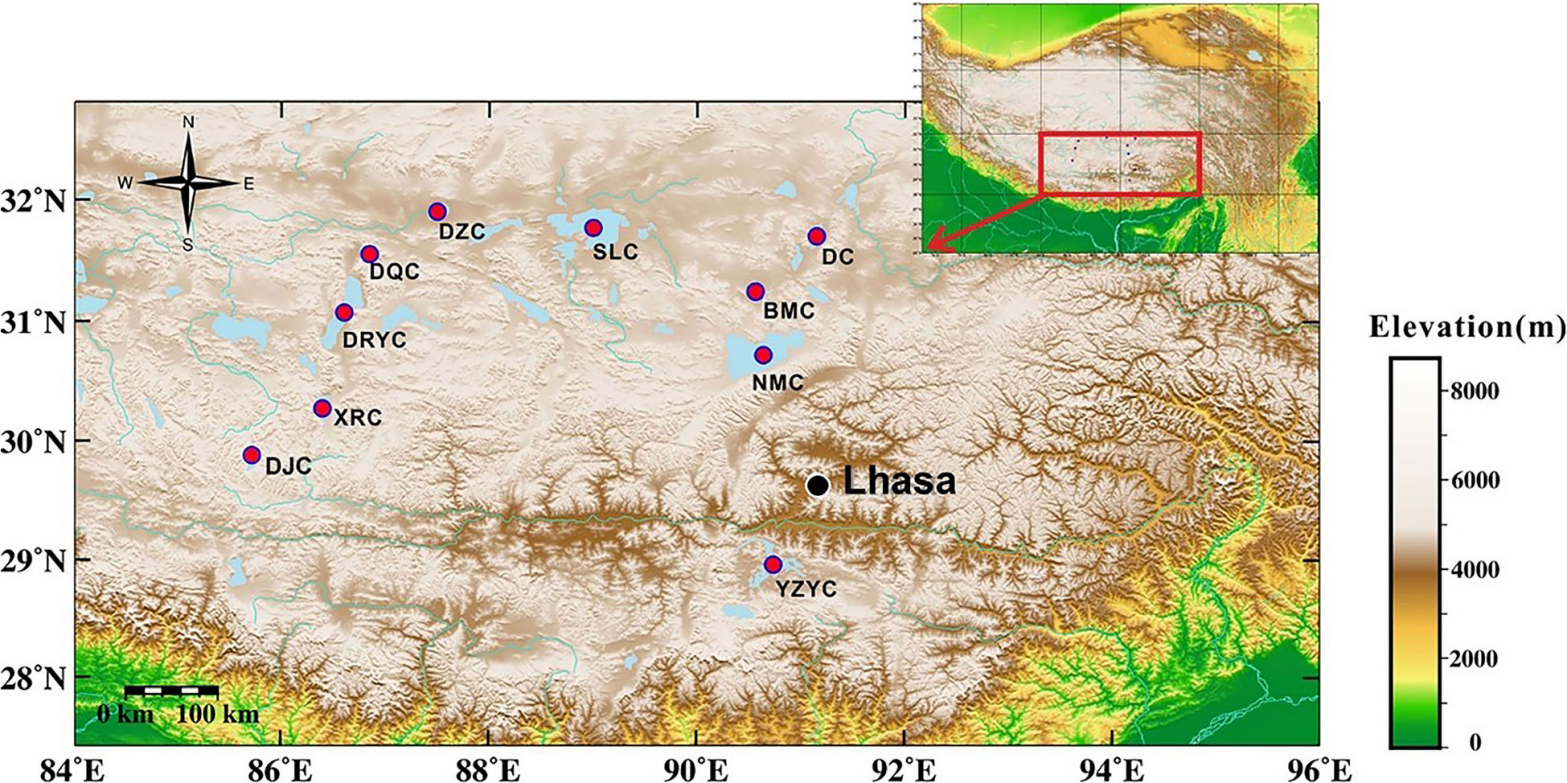
Map of Tibetan saline lakes sampled for this study. NMC, Namucuo Lake; SLC, Selincuo Lake; DRYC, Dangreyongcuo Lake; YZYC, Yangzhuoyongcuo Lake; BMC, Bamucuo Lake; DZC, Dazecuo Lake; DQC, Dangqiongcuo Lake; DJC, Dajiacuo Lake; XRC, Xurucuo Lake; DC, Dongcuo Lake

A total of 31 sediment samples were collected from ten lakes on the Tibetan Plateau between September 5 and 18, 2015, including Namucuo (NMC), Selincuo (SLC), Dangreyongcuo (DRYC), Yangzhuoyongcuo (YZYC), Bamucuo (BMC), Dazecuo (DZC), Dangqiongcuo (DQC), Dajiacuo (DJC), Xurucuo (XRC), and Dongcuo (DC), with 2 to 8 samples per lake (Fig. 1). The sampling areas were located between elevations of 4,406 to 5,166 m. The hydrological connectivity has been examined by Google Earth imagery as previously described [22]. Lake salinities ranged from 0.25 to 123.28 g/L.

The surface sediment samples (0–5 cm), with variable proportions of sand and mud, were collected near the lake edges on different sites around the lakes, each corresponding to a sample. The soil auger set was used to collect sediments. For each sample, we collected five replicates and mixed thoroughly in situ. The composite sediments were stored in sterilized 50-ml tubes and frozen immediately in dry ice for transport back to the lab. Samples for microbial and geochemical analysis were stored at − 80 °C until further analysis.

### Environmental parameters determination

Lake water pH was measured in triplicate in situ by pH probe V3.0 (Atlas Scientific) at the time of sampling. Concentrations of major cations and anions ( F^−^, Cl^−^, NO_2_^−^, SO_4_^2+^, Br^-^, NO_3_^−^, Li^+^, Na^+^, and K^+^) were measured using ion chromatography (Dionex, ICS-2100). Total organic carbon (TOC) and total nitrogen (TN) were measured by CN Analyzer (Vario Max CN, Elementar, Germany). GPS coordinates were recorded at each sampling point, ranged from 28° 58′ 29.53″ N to 31° 49′ 19.45″ N and 85° 44′ 57.78″ N to 91° 06′ 49.54″ N (Additional file 1: Table S1). The mean of lake water temperatures for the month (August 2015) prior to sampling dates was extracted from public data [23].

### epicPCR and 16S rRNA gene amplicon PCR library preparation

The epicPCR procedure was performed following published protocols [19] with minor modifications. Briefly, the sediment samples were suspended in sterile water and mixed by shaking (30 min, 720 rpm; IKA® KS 130 B, Staufen, Germany) and sonication (1 min, 20 W; Microson XL2000 ultrasonic liquid processor with 1.6 mm diameter micro-tip probe, Misonix, NY, USA) [24], followed by filtration of the bacterial suspension through a 35-μm cell strainer to remove sedimentary particles. The cell density was determined by fluorescence microscopy after fixation with formalin and DAPI (4′,6-diamidino-2-phenylindole) staining. The suspension was adjusted to 10–20 million cells per 30 μl (volume used for reaction per sample) and subjected to polymerization by acrylamide/BAC (N,N′-Bis(acryloyl)cystamine, Sigma) and ammonium persulfate in STT emulsion oil (4.5% Span 80, 0.4% Tween 80, 0.05% Triton X-100, mixed by *v*/*v* in mineral oil). TEMED (N,N,N′,N'-Tetramethylethylene-diamine) was added as a catalyzing agent. The polymerized acrylamide beads were then extracted with diethyl ether, resuspended in 1× TK buffer (20 mM Tris-HCl (pH 7.5), 60 mM KCl), and filtered through a 35-μm cell strainer for subsequent fusion PCR. The validation of single cell separation was verified by fluorescence microscope observation. Then, fusion PCR amplification was conducted in ABIL emulsion oil (4% ABIL EM 90, 0.05% Triton X-100, mixed by *v*/*v* in mineral oil). The thermal cycling conditions were as follows: an initial denaturation at 94 °C for 30 s, followed by 33 cycles at 94 °C for 5 s, 52 °C for 30 s, and 72 °C for 30 s, and ended with a final extension step of at 72 °C for 5 min. Immediately after the run, the products were extracted with diethyl ether and ethyl acetate. Agencourt AMPure XP - PCR Purification (Beckman Coulter, Danvers, MA, USA) beads were then used for product purification. The purified products were used as template for the following nestPCR reaction. Nested qPCR was performed on each sample with 100× SYBR Green I (Invitrogen, Waltham, MA, USA) to determine optimal nested PCR cycle numbers for different samples accompanied by a non-template negative control. The Ct values were used to dilute each sample to approximately the same concentration of fusion products. The nestPCR conditions were as follows: 98 °C for 30 s, 40 cycles of 98 °C for 5 s, 52 °C for 30 s, 72 °C for 30 s with final extension at 72 °C for 5 min. The product fragment sizes were checked by agarose gel electrophoresis and then harvested and purified using Gel Extraction Kit (D2500-02, OMEGA BioTek) for library construction. The library was prepared according to the MiSeq Reagent Kit Preparation Guide (Illumina 250 bp – 250 bp) and sequenced on an Illumina MiSeq platform. Primers dsrB-F1, R2 (1492R), and dsrB-R1_519R were used for fusion PCR, and primer set dsrB-F3/E786R with attached barcodes plus blocking primer set U519F-block10/U519R-block10 were used for nestPCR (Additional file 1: Table S2 and S3) [19].

For 16S rRNA gene amplicon sequencing, DNA from each sample was extracted with FastDNA^TM^ SPIN Kit for Soil (MP Biomedicals). DNA quality and concentration were determined by a NanoDrop Spectrophotometer (Nano-100, Aosheng Instrument Co Ltd.). The 16S rRNA gene was amplified with the universal pair of primer 515F (5′-GTGCCAGCMGCCGCGGTAA-3′) and 806R (5′-GGACTACHVGGGTWTCTAAT-3′) combined with self-designed barcodes to distinguish samples (Additional file 1: Table S3). The PCR reaction consisted of 0.5 μLTaq DNA Enzyme (TaKaRa), 5 μL 10 × PCR buffer, 1.5 μL 10 μM dNTP mixture, 1.5 μL of both 10 μM forward and reverse primers, 1 μL of template DNA within 20–30 ng/μL, and ddH2O adjusted to a final volume of 50 μl. The following program was used: denaturation at 94 °C for 1 min, 30 cycles of 94°C for 20 s, 57 °C for 25 s, and 68 °C for 45 s, final extension at 68 °C for 10 min followed by sample maintenance at 4 °C. The final DNA purification, library construction, and sequencing procedures were exactly the same as that for epicPCR products.

### epicPCR and 16S rRNA gene amplicon sequence processing

Sequence processing was performed using an in-house pipeline [25] that integrated various bioinformatics tools. Raw sequencing reads of both epicPCR products and 16S rRNA amplicons were assigned to samples according to their barcodes, allowing for one mismatch. Both forward and reverse primers were trimmed, also with one mismatch allowed. Paired-end reads of sufficient length (> 200 bp) of 16S rRNA amplicons with a minimum 30 bp overlap were combined by FLASH program [26] into full-length sequences, resulting in an average fragment length of 253 bp. For epicPCR reads, the steps for barcode and primer removal and FLASH were similar, except the average fragment length was 283 bp. After FLASH, the beginning 30 bp of each sequence from epicPCR reads, the *dsrB* gene fragments connected to the corresponding 16S rRNA gene sequences, were removed before subsequent processing. The bridging primer within epicPCR reads was then trimmed after the FLASH step, resulting in 16S rRNA fragments and dsrB fragments. The Btrim program [27], with a threshold of quality score > 20 and a window size of 5, was used to filter out unqualified sequences of the 16S rRNA fragments from epicPCR and 16S rRNA amplicons, respectively. Any sequences with either an ambiguous base or beyond the length range of 245~260 bp were discarded. The 16S rRNA amplicon sequences and 16S rRNA fragments from epicPCR reads were then combined for subsequent analysis. After filtering the sequences for 16S rRNA amplicons and epicPCR reads were then combined into one sequence file (fasta format) by an in-house pipeline. Thereafter, UPARSE [28] was used to remove chimeras and classify the sequences into operational taxonomic units (OTUs) at 97% sequence identity without any singletons being discarded. Resampling to 10,000 reads per sample was used to normalize total reads across samples. Selected representative OTU sequences were aligned using PyNAST [29] and tree file was generated using FastTree program [30]. The OTU tables of 16S rRNA sequences that support the findings of this study are available on the institute’s website through the following link, http://mem.rcees.ac.cn/download.html. The raw sequencing data have been deposited in the NCBI Sequence Read Archive under accession code PRJNA489931 (Additional file 1: Table S4).

Shannon indices of α-diversity were used to quantify the biodiversity of microbial community or SRP. Richness was obtained by counting the observed OTUs numbers in resampled OTU table. Chao1 values [31] associated with the rarefaction curve were calculated using mothur program [32]. Shannon index and correlation of α-diversity index were calculated according to species abundance using vegan package (v.2.3-5) in R (v.3.2.5) [33].

### Sequence alignment and phylogenetic analysis

The highly abundant (> 1% in at least one sample) OTUs of SRP extracted from epicPCR sequencing were aligned with reference sequences of the relevant phyla acquired from NCBI for phylogenetic positioning using MUSCLE with default settings [34]. The aligned sequences were processed through the Phlogeny.fr pipeline [35] for maximum-likelihood tree construction and interactive tree of life (iTOL) v3 [36] for tree visualization. The bootstrap supported ≥ 50% by 1000 resamplings were indicated on the tree.

### Statistical analysis

Most statistical computations, including PCoA analysis, were run on an in-house pipeline (available at http://mem.rcees.ac.cn:8080) to generate β-diversity between lakes, and Mantel test for the assessment effects of environmental factors. Three correlation tests (Pearson, Kendall, and Spearman) were applied to test the Shannon index for any correlation between SRP and microbial community α-diversity. Mantel test was performed to verify the associations between microbial community and environmental variables, for which the Jaccard distances were calculated for microbial and SRP communities across 31 samples and the Euclidean distances used to measure the variation of standardized environmental variables across samples. The results from the Mantel test were further validated by canonical correlation analysis (CCA).

## Results

### The environmental conditions of sampling lakes

A wide range of environmental factors was measured across all samples. At the ten sampled lakes, pH ranged from 8.86 ± 0.02 to 9.68 ± 0.01, salinity from 0.44 ± 0.14 to 122.39 ± 1.26 g/L, total nitrogen (TN) from 0.07 ± 0.03 to 0.88 ± 0.31 g/kg, total organic carbon (TOC) from 0.11 ± 0.02 to 2.08 ± 1.09%, and sulfate concentration from 0.15 ± 0.04 to 5.25 ± 0.11 g/L (Additional file 1: Table S5). The mean of lake water temperatures for the month prior to sampling dates was extracted from public data, ranging from 9.95 to 14.75 °C [23].

### The composition of microbial communities and SRP sub-communities shows endemism

**Fig. 2:**
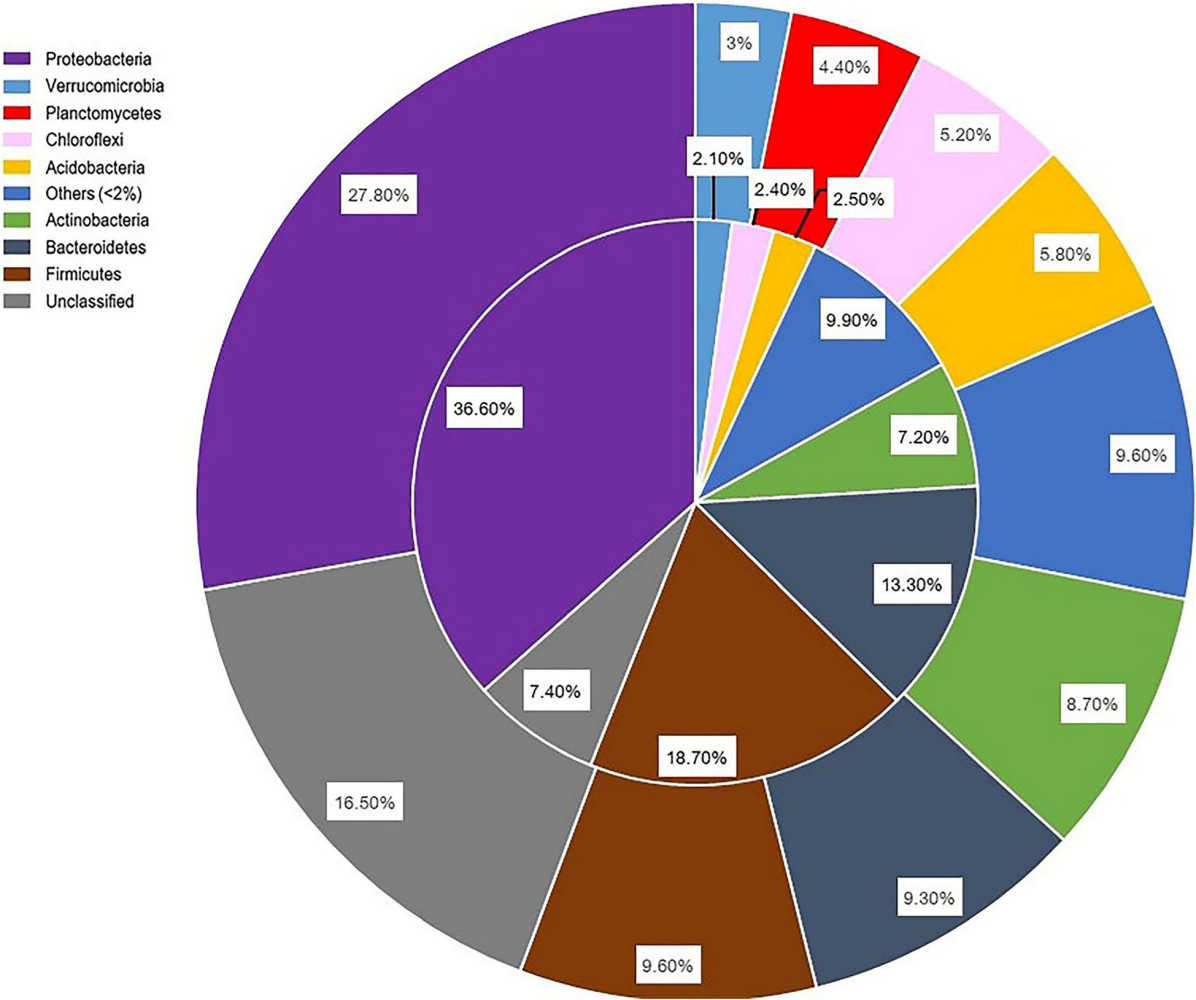
The percentage of representative OTUs affiliated with the major phyla of the microbial community and SRP as identified by 16S rRNA amplicons (outer pie) and epicPCR (inner pie), respectively

The compositions of the entire microbial (i.e. bacterial and archaeal) community and SRP sub-community were profiled by sequencing of 16S rRNA gene amplicons and epicPCR (16S rRNA+*dsrB* gene) amplicons, respectively, from 31 samples (Table 1). After removing barcodes and quality control, all epicPCR reads were trimmed on the bridging primer and split into 16S and *dsrB* files (Additional file 1: Table S6). After completion of preprocessing, a total of 1,350,398 and 622,205 reads were generated by 16S rRNA gene amplicons for the entire microbial community and epicPCR for SRP, respectively. The sequences of 16S rRNA fragments from epicPCR and 16S rRNA genes for the entire microbial community were used for all below analyses. For the entire community, there were 12,519 representative OTUs affiliated with 890 genera and 42 phyla. For the SRP sub-community, there were 883 representative OTUs affiliated with 230 genera belonging to 30 phyla with 120 OTUs with high relative abundance (> 1% in at least one sample). Within those highly abundant SRP-OTUs, 113 belonged to 60 known genera in 16 bacterial phyla (mainly *Proteobacteria, Firmicutes*, and *Bacteroidetes*) and one archaeal phylum (SRP-OTU111, genus *Nitrososphaera*, phylum *Thaumarchaeota*); in addition, there were 7 unclassified OTUs (1 archaeal and 6 bacterial). To demonstrate the taxonomic composition across the main phyla, OTU numbers affiliated with each phylum were compared between the entire microbial community and SRP sub-community. *Proteobacteria* (27.8% and 36.6%, in the entire community and SRP, respectively), *Firmicutes* (9.6% and 18.7%), and *Bacteroidetes* (9.3% and 13.3%) were the most diverse phyla, at OTU level, accounting for the largest proportion of OTU numbers in all samples (Fig. 2). Across different lakes, *Proteobacteria* and *Firmicutes* were the most abundant phyla for the entire community, while *Proteobacteria* was always the most abundant phylum for the SRP sub-community (Additional file 1: Figure S1).

**Table 1:** Comparison of composition of microbial community and SRP populations at OTU, genus, and phylum levels in Tibetan lakes

	Microbial community	SRP sub-community	High abundant SRP (> 1% in at least one sample)
OTUs	12,519	883	120
Genus	890+ unclassified	230+ unclassified	60+ unclassified
Phylum	42+ unclassified	30+ unclassified	17+ unclassified

For microbial communities as a whole and SRP sub-communities, the majority of the representative OTUs showed endemism (Additional file 1: Table S7). Less than 0.6% of the total microbial community OTUs (74 out of 12,519) were ubiquitous, present in each lake, while 67.5% (8,463 of 12,519) of the OTUs were lake-specific. For SRPs, nearly 70% OTUs (615 out of 883) were lake-specific while 2% (18 out of 883) were ubiquitous. Of the 120 SRP-OTUs, the majority (> 75%) were found to be lake-specific, as they were observed in high relative abundance in only single lake, and only two SRP-OTUs (SRP-OTU1 affiliated with *Halomonas*) and (SRP-OTU8 affiliated with *Shewanlla*) were present in high relative abundance in all lakes. As Tibetan saline lakes are considered an extreme environment for isolating new microorganisms, the proportions of unclassified taxa in both the entire microbial community and SRP sub-community were compared at the genus level (Additional file 1: Figure S2). At the genus level, the unclassified proportion of the whole microbial community was higher than that of SRP sub-community, suggesting the microbial community is a potentially rich source for uncharacterized microbial strains; however, in comparison, the SRP group had a smaller proportion of unknown species. Similarly, at the OTU level, unclassified organisms accounted for 7.4% of OTUs in SRP, while in the entire microbial community, they accounted for 16.5% (Fig. 2).

### The comparison of α- and β-diversity in microbial entire communities and SRP sub-communities

**Fig. 3:**
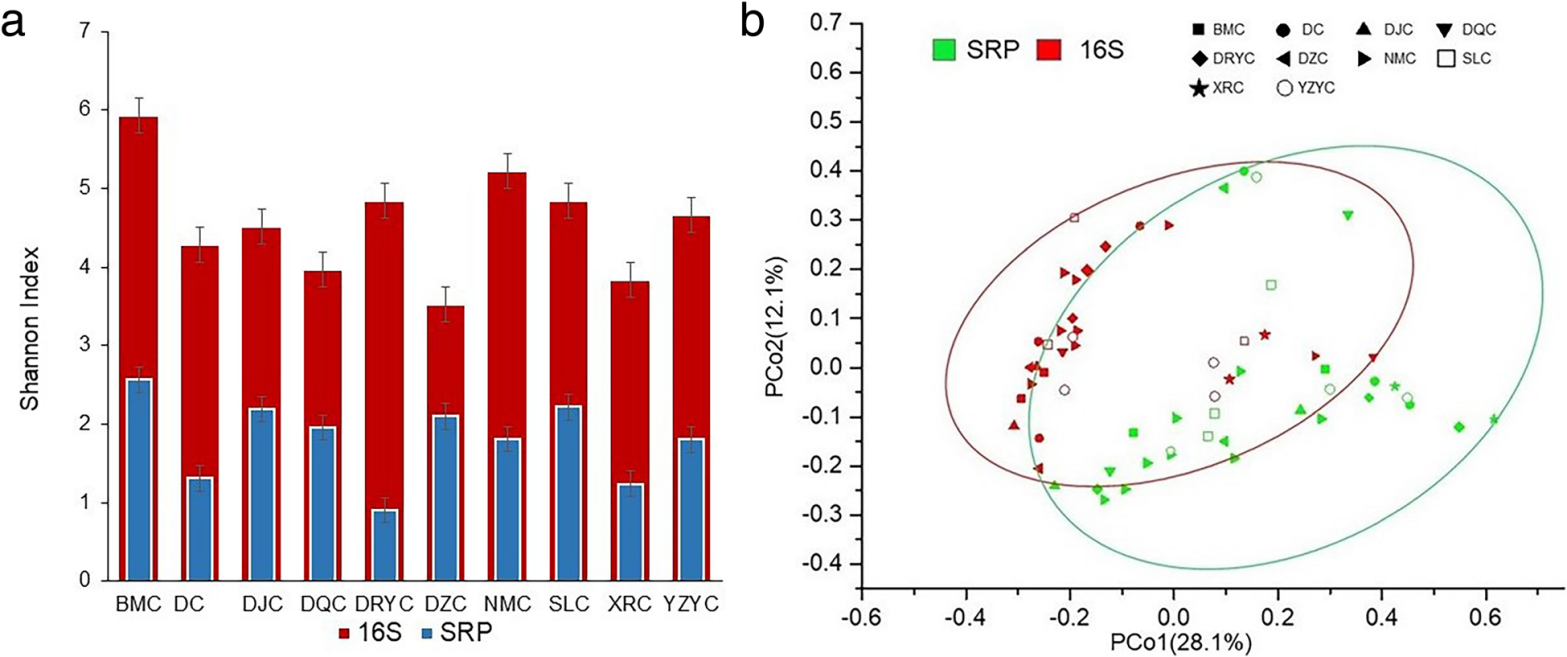
The diversity and structure of microbial and SRP communities in Tibetan saline lakes. **a** The α-diversity i (Shannon index) of microbial community and SRP in each lake. The lakes were ordered according to their salinity from lowest (NMC) to highest (DQC). The error bars indicate standard deviation. **b** The PCoA plot for Unifrac distances of microbial community and SRP sub-community

The α- and β-diversity of microbial communities and SRP sub-communities in each lake were assessed for their associations (Fig. 3). The α-diversity measured by the Shannon indices is the numeric indication for each samples’ species richness [37], while β-diversity provides regional diversity from compositional differences between site assemblages [38] which, here, is described by PCoA analysis. The Shannon diversity indices were calculated at the OTU level for each sample, ranging from 3.53 to 5.92 for the entire microbial community and from 0.90 to 2.56 for SRP (Fig. 3a). The correlation coefficients of Shannon diversity indices between microbial communities and SRP sub-communities were 0.443 (*P* < 0.05) by Pearson’s correlation. The β-diversity ordination analysis of PCoA (unifrac weighted) showed an overlap in the distribution between the entire microbial community and SRP populations in the majority of samples (Fig. 3b), with the two axes explaining 40.2% of the total variation. The PCoA analysis (unifrac weighted) on β-diversity showed a larger dispersal for the SRP sub-communities than for the whole microbial communities (Fig. 3b), possibly due to there being fewer core OTUs in SRP than microbial communities across all sampled lakes.

### The phylogenetic position of SRP genera of high relative abundance

**Fig. 4:**
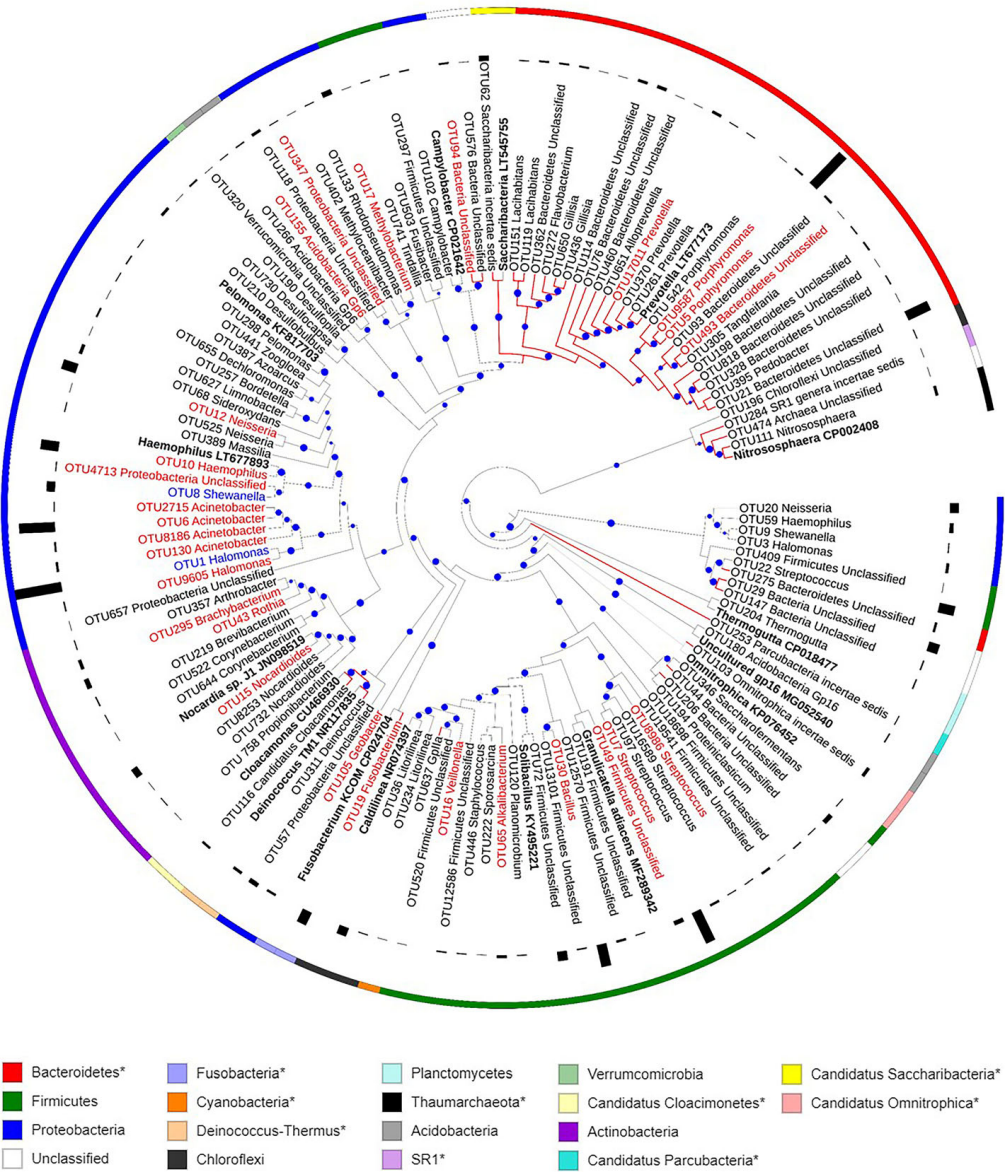
The phylogenetic positions of high-abundance OTUs classified as SRP by epicPCR. Fonts in bold indicate reference sequences, those in blue indicate the core OTUs (high abundance in at least one sample from any lake), in red indicate non-specific OTUs (high abundance in samples collected from at least two lakes), and in black indicate lake-specific OTUs (high abundance in samples from a single lake). Red arc lines indicate OTUs affiliated with the 11 phyla previously unreported to be involved in dissimilatory sulfate reduction. The size of blue circles on branches represent the bootstrap values of 50–100. The bars around the tree indicate the relative abundance of OTUs in the lake where they were most abundant. The outer ring of strips is the taxonomic affiliation of OTUs at the phylum level. The legend on the bottom indicates the colors used for phyla in the outer strip

A maximum likelihood phylogenetic tree of the 120 highly abundant (> 1% in at least one sample) SRP-OTUs was constructed to show the phylogeny, abundance, distribution, and taxonomy of the SRP in Tibetan saline lakes (Fig. 4). SRP-OTU1 (genus *Halomonas*; phylum *Proteobacteria*) was, on average, the most abundant in all lakes. The 16 observed bacterial phyla included 7 (*Proteobacteria*, *Firmicutes*, *Acidobacteria*, *Actinobacteria*, *Chloroflexi*, *Planctomycetes*, and *Verrucomicrobia*) known to be capable of sulfate/sulfite reduction [18]. Additionally, there were 42 OTUs affiliated with 9 bacterial phyla, including four candidate phyla, one archaeal phylum, and several unclassified SRP, that did not contain microorganism known for sulfate/sulfite reduction function, nor previously known to possess sulfate/sulfite reductase genes. Among the phyla previously unknown to possess sulfate/sulfite reductase genes, *Bacteroidetes* had the most diverse OTUs, these were clustered into a single sub-clade on the tree.

### The dominated environmental factors shaping the SRP and microbial community

Mantel tests were used to assess the effect of environmental factors on the composition of both the entire microbial communities and SPR sub-communities. For microbial community, pH (*r*_M_ = 0.199, *P* = 0.004) and mean water temperature during the month prior to sampling (August) (*r*_M_ = 0.329, *P* = 0.003) exhibited the most significant associations with the community variations across the 31 sedimentary samples (Table 2). However, for the SRP sub-community, the mean water temperature (*r*_M_ = 0.131, *P* = 0.044) and total nitrogen (*r*_M_ = 0.388, *P* = 0.016) were significantly associated with their sub-community structure. Again, for the 120 high-abundance SRP populations, the mean water temperature (*r*_M_ = 0.135, *P* = 0.044) and total nitrogen (*r*_M_ = 0.351, *P* = 0.021) exhibited significant effects. Total nitrogen (*r*_M_ = 0.158, *P* = 0.047) was the only environmental factor detected as being significant for lake-specific SRPs. The significant environmental factors for each group were further assessed by CCA analysis in order to validate the results (Additional file 1: Figure S3). The results from CCA analyses (Additional file 1: Table S8) verified pH significant for the microbial community (*F* = 1.49, *P =* 0.002) and total nitrogen significant for both SRP sub-community (*F* = 1.89, *P =* 0.03) and high-abundance SRP population (*F* = 1.98, *P =* 0.02). Together, these results indicated that the environmental factors involved in shaping the entire microbial community and SRP sub-community were substantially divergent.

**Table 2:** Simple Mantel test (Jaccard distance) of environmental factors on microbial community and SRP sub-community

	Microbial community		SRP sub-community		Highly abundant SRP populations		Lake-specific SRP populations	
	*r*_*M*_	*P*	*r*_*M*_	*P*	*r*_*M*_	*P*	*r*_*M*_	*P*
pH	0.1993	*0.004*	0.0381	0.311	0.0529	0.24	0.0248	0.351
MMT(Aug)	0.3299	*0.003*	0.1314	*0.044*	0.1349	*0.044*	0.0495	0.198
TN	− 0.0869	0.759	0.3878	*0.016*	0.3513	*0.021*	0.1580	*0.047*
TOC	0.0805	0.224	− 0.1159	0.807	− 0.0991	0.821	− 0.0112	0.532
SO_4_^2−^	0.1364	0.095	0.0802	0.205	0.0521	0.251	− 0.0349	0.660
Salinity	0.0125	0.371	− 0.0313	0.491	− 0.0858	0.771	− 0.0895	0.796

The *P* values of statistical significance (< 0.05) are in italics.

*Abbreviation*: *MMT (Aug)* monthly mean temperature of surface water in August, 2015, *TN* total nitrogen, *TOC* total organic carbon, *SO*_*4*_^*2−*^ sulfate concentration

## Discussion

Understanding the roles of microbial groups with ecologically important functions, such as SRP, in various ecosystems has largely relied on bringing organisms into culture and assessing their metabolic capabilities. In recent years, metagenomics and single-cell genome sequencing have been frequently applied to connect taxonomy with these key ecological functions and attempted to assess the diversity of functional taxa in their natural habitats [39–41]. The diversity of the SRP functional taxa has been confined to a restricted range of microbial phyla for a number of years [42], partly due to a lack of valid detection technologies. In 2018, a study by functional computation on public metagenomic data detected many novel SRP phyla, suggesting a tremendous underestimation of the extent of SRP diversity by routine surveys on rRNA or dsrAB genes. This study proposed a deeper mining of available microbial genomes from public databases for exploring candidate sequences of sulfate/sulfite reducing functional genes so as to reveal more SRP taxa [18]. Herein, our study used epicPCR to investigate into the SRP sub-community composition of ten Tibetan saline lakes at a regional scale, through which ten phyla previously unknown for their potential dissimilatory sulfate/sulfite reduction function have been discovered. Additionally, the high-resolution structure of the SRP sub-community enables an overview of its diversity, comparison to the entire microbial community, and elucidation of effective environmental determinants. Our results indicated that a far more diverse range of microorganisms is involved in sulfate/sulfite reduction than previously realized.

In the 31 samples collected, *Proteobacteria*, *Firmicutes*, and *Bacteroidetes* were the most diverse phyla with the highest percentages of representative OTUs. These phyla are commonly reported in saline lake environments and several subgroups are known to be moderate or extreme halophiles [43]. These phyla are also dominant in a number of alkali lake sediments across the Tibetan region [14], demonstrating their wide presence in analogous ecological habitats. It is believed that these phyla are frequently detected due to their ability to adapt to the harsh conditions found in different lake types [44]. At the genus level, *Halomonas* and *Acinetobacter* were the most abundant genera in Tibetan saline lakes. While the microbial population of these lake sediments is largely uncharacterized, the majority of SRP belonged to well-studied taxa, which may be partly due to the primers used for amplification of *dsrB* in epicPCR.

However, we also found the endemism is a feature in both the whole microbial communities and the SRP sub-communities within the ten lakes we studied here (Additional file 1: Table S7). The distant locations for these lakes and lack of structural connectivity between them are clear and may be a contributing factor to the endemism as microbial dispersal limitation can be constrained and the successional trajectory and assembly of microbial communities could be different. It has been previously observed that the lack of hydrological connectivity can give rise to the emergence of different microbial taxa in disconnected lakes in response to glacier runoff [45], indicating the disconnection enables lakes to undergo different environmental transformations, causing a divergence between their microbial communities.

Among the 120 highly abundant SRP-OTUs from epicPCR sequencing, members of the *Proteobacteria* (35 SRP-OTUs), *Firmicutes* (25 SRP-OTUs), *Acidobacteria* (13 SRP-OTUs), *Verrucomicrobia* (SRP-OTU320 unclassified *Verrucomicrobia*), *Actinobacteria* (10 SRP-OTUs), *Planctomycetes* (SRP-OTU204 *Thermogutta*), and *Chloroflexi* (4 SRP-OTUs) have all been noted for their ability to reduce sulfate/sulfite [18]. Also of note, the sequences included two SRP-OTUs from *Archaea*, SRP-OTU111 *Nitrososphaera* and SRP-OTU474 unclassified *Archaea*. While in principle epicPCR primers target bacterial but not archaeal *dsrB*, a speculation for the occurrence of these archaeal sequences is low phylogenetic information available for the *dsrB* fragment [19]. More than 90 of the 120 SRP-OTUs are lake-specific, while only 2 are widely distributed in every lake, suggesting the majority of SRP taxa may have high sensitivity to environmental conditions. It revealed that they can only adapt to and emerge in specific lakes, or alternatively, this phenomenon may be attributed to the various successional trajectory for the SRP sub-community assembly. In the two ubiquitous SRP-OTUs, the abundant SRP-OTU1 affiliates with *Halomonas*, a halophilic genus involving in the sulfur cycle [46], though its exact role in sulfate/sulfite reduction awaits further study. In our study, *Halomonas* is the most abundant genus within the SRP group, present in high relative abundance in every lake. This is likely due to *Halomonas* spp. tolerance of high salinity conditions [47]. Beyond the known SRP, 21 SRP-OTUs belonged to 14 genera with no previously documented ability for sulfate/sulfite reduction (Fig. 4). However, the genomes of *Halomonas* strains are found to include *dsr* family genes, for instance, genes HAL1_10182 of *Halomonas* sp. HAL1 [48] and MOY_00165 in GFAJ-1 [49] encoding dissimilatory sulfite reductase subunits, supporting their potential involvement in this activity. Within the OTUs affiliated with new discovered phyla, the majority of *Bacteroidetes* clustered with *Candidatus Saccharibacteria* into a subclade, while similarly Candidate Division SR1 and *Thaumarchaeota*, *Candidatus Cloacimonetes*, and *Deinococcus-Thermus* formed subclades. Most of these subclades from candidate taxonomies implied that there are still a number of unknown taxa potentially possessing sulfur reducing capabilities.

To elucidate the deterministic environmental factors influencing the community assembly within Tibetan saline lakes, Mantel tests were performed on the entire microbial community, SRP sub-community, high abundance SRPs, and lake-specific SRPs. The monthly mean temperature (MMT) of August 2015 in lake surface water and pH significantly affected the microbial community composition (*P* < 0.005), while MMT and TN shaped the SRP sub-community (Table 2). For lake-specific SRPs, TN was the only significant environment factor, indicating their endemism may be either influenced by some unknown environmental factor or successional trajectory of sub-community assembly. The environmental factors of significance were further validated by CCA analysis (Additional file 1: Figure S3). The *P* values of CCA results verified the pH as significant for the entire microbial community and TN for SRPs. Previous studies have demonstrated that mean annual temperature (MAT) was the most important environmental factors shaping both α- and β-diversities of microbial communities in forest soils [50–52]. In a tropical freshwater lake, the monthly mean temperature of water was discovered to be a key factor for phytoplankton biomass through linear regression. Similarly, the monthly values of sea surface water temperature had a significant relationship with bacterial production and alkaline phosphatase activity in marine ecosystems [53]. Our results indicated that the monthly mean temperature of in situ water in addition to local atmosphere temperature can alter the microbial community structure in lake environments, implying the important effect of long-term temperatures. Mantel test results also showed pH as another significant factor for shaping the entire microbial community in saline lakes. The significance of pH on microbial community in lake sediments from Tibetan plateau has been noted previously [14, 54]. As soil and sediments have similar nutrient compositions including nitrogen, carbon, and phosphorus [55], pH has previously been shown to have significant effects on microbial communities at regional and continental scales of soils in Britain, France, and North America [56–58]. It is also a significant driver for shaping the microbial community structure in sediments collected from Lake Hazen, located in the Canadian Arctic, which is an extreme environment [59] as well as in sediments from typical freshwater ecosystems of the Qiantang River [60]. Notably, our lake water samples showed a narrow range of pH values (8.86 ± 0.02 to 9.51 ± 0.01, Additional file 1: Table S5), but the sediment microbial community still demonstrated a high responsiveness to even subtle shifts in pH. Apart from MMT, TN showed significant associations for SRP sub-communities according to both Mantel Test and CCA results. TN has been shown to impact microbial community structure and SRPs diversity in soil, and in aquatic microcosms, putatively through acidification and depletion of oxygen and hitherto changing in situ microbial metabolisms [61, 62]. Our results by dual methods demonstrated that the temperature and TN content are the major drivers on shaping SRP sub-community in saline lakes and could be different from the environmental drivers for the entire community.

Our study also showed there were divergent environmental drivers for SRP sub-community and entire microbial community across ten saline lakes (Table 2). In natural sediments, SRPs account for only a small portion of the entire microbial community, as exemplified by 0.03–0.28% in sediments from north China marginal seas [63] or 1.4 to 15% in sediments collected from Aarhus Bay [64]. Therefore, the SRP populations’ variations caused by specific environmental drivers will not necessarily shift the entire microbial community at a statistically significant level. The other microbial community members, other than SRPs, can be sensitive to some common environmental drivers, such as pH (Table 2).

The positive correlation of α-diversity between microbial communities and SRP sub-communities implies more diverse microbial consortia have SRPs with higher species richness and thus increased functional redundancy. Although the microbial communities and SRPs showed endemic feature in these lakes, their variations do not excluded the potential for functional redundancy of sulfate-reduction, as indicated by widespread presence of the *dsrB* gene within genomes from across a broad range of phyla, implicating an active sulfur biogeochemical transformation cycle maintained by functional groups despite fundamentally differences between the taxa present. We discovered more taxonomically distinct prokaryotes than previously recognized that encode the same sulfate-reducing function. The functional redundancy and taxonomical diversity may insure sulfate-reducing activity against alteration in community composition and diversity [65]. The phenomenon that taxonomically different microbes performing the similar functions successfully colonize niches was postulated as that functionally equivalent species can occupy the same niche, but the first-arriving species ultimately establishes itself within communities [66], which may provide a clue to track the taxonomic source of the SRP sub-community across the lakes.

## Conclusion

For several decades, sulfate-reducing prokaryotes have been recognized as functional microorganisms present in various environments, but their full taxonomic distribution, especially in the natural ecosystem, is still unclear, largely due to the limitation of available technologies. We applied a newly developed approach, epicPCR, to probe the sulfate-reducing prokaryote community from Tibetan saline lake sediments, and assigned the taxa of SRP populations to 17 highly abundant phyla, including ten with previously unrecognized sulfate-reducing taxa in this unique habitat. Observing SRP biogeography at regional scales, separate from the rest of the microbial community, allowed a better understanding of their diversity, distribution, and deterministic environmental factors. Our results showed that the structural shifts of microbial communities as a whole, and SRP sub-communities alone, varied in different ways and in response to different environmental factors with the SRP taxonomic richness. It implied that the Tibetan saline lakes contain a large inventory of uncharacterized SRP. Our study further provided a starting point for detecting SRP from such extreme environments at high resolution and comprehensively constructing a database for the distribution and taxonomical analysis of SRP. As biotic interactions between functional community members within microbiomes are important, it is also necessary to construct the interaction network between SRP group with other functional groups, such as nitrogen and carbon cycling groups and the rest of the microbial community to generate versatile models on biogeochemical functions. Finally, our study proved that epicPCR could be a robust approach for detecting SRP populations from various environmental niches, including both natural ecosystems as well as artificial systems such as industrial bioreactors.

## Additional file

Additional file 1: **Figure S1.** The relative abundance for each lake at phylum level. **Figure S2.** The relative abundance of unclassified genera in the whole microbial community and SRP sub-community. **Figure S3.** CCA analysis. **Table S1.** The geographical features of the saline lakes. **Table S2.** Primers for epicPCR and 16S rRNA gene PCR amplification. **Table S3.** barcodes for epicPCR and 16S rRNA amplicons. **Table S4.** Accession number for Biosamples in NCBI. **Table S5.** The environmental factors of the saline lakes. **Table S6.** The number of remained epicPCR reads for the quality checking processing. **Table S7.** The distribution of core and lake-specific OTUs shows endemism for microbial communities and SRPs across lakes. **Table S8.** F and P values of CCA analysis. (DOCX 346 kb)
