# Supplementary material for: Unraveling the diversity of sedimentary sulfate-reducing prokaryotes (SRP) across Tibetan saline lakes using epicPCR
Source: Microbiome. 2019 May 4;7:71. doi: 10.1186/s40168-019-0688-4 (PMC6500586; doi:10.1186/s40168-019-0688-4)
Supplement: Supplementary file 1 — Figure S1. The relative abundance for each lake at phylum level. Figure S2. The relative abundance of unclassified genera in the whole microbial community and SRP sub-community. Figure S3. CCA analysis. Table S1. The geographical features of the saline lakes. Table S2. Primers for epicPCR and 16S rRNA gene PCR amplification. Table S3. barcodes for epicPCR and 16S rRNA amplicons. Table S4. Accession number for Biosamples in NCBI. Table S5. The environmental factors of the saline lakes. Table S6. The number of remained epicPCR reads for the quality checking processing. Table S7. The distribution of core and lake-specific OTUs shows endemism for microbial communities and SRPs across lakes. Table S8. F and P values of CCA analysis. (DOCX 346 kb) [file 40168_2019_688_MOESM1_ESM.docx]

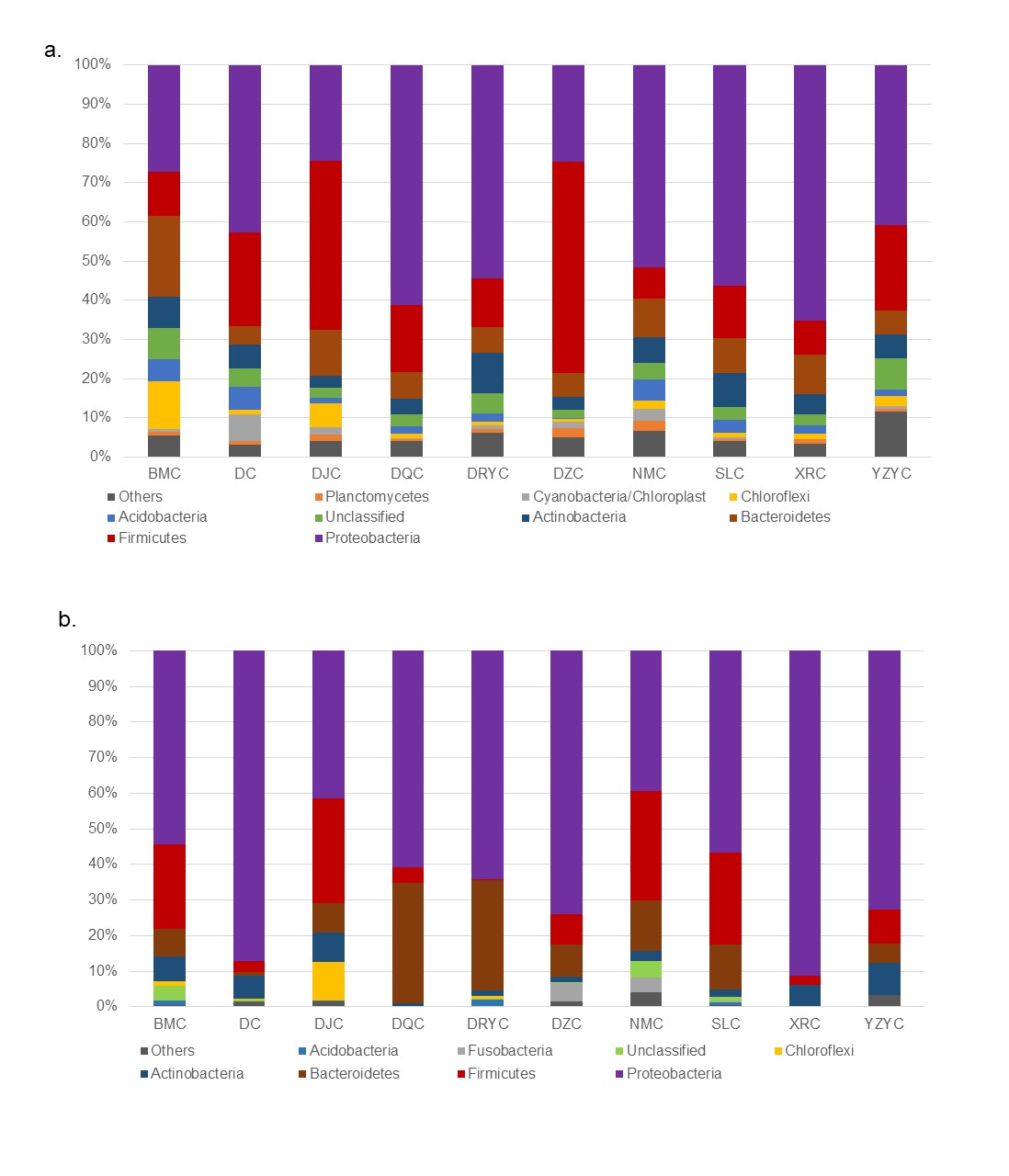


Figure S1. The relative abundance for each lake at phylum level of whole microbial community (a) and SRP sub-community (b).


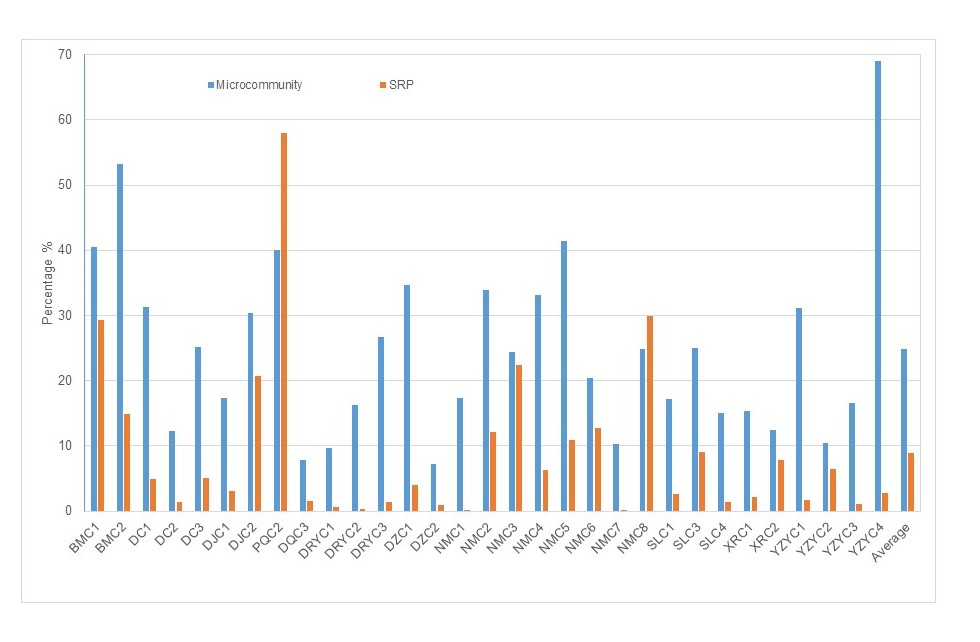


**Figure S2.**The relative abundance of unclassified genera in the whole microbial community and SRP sub-community.


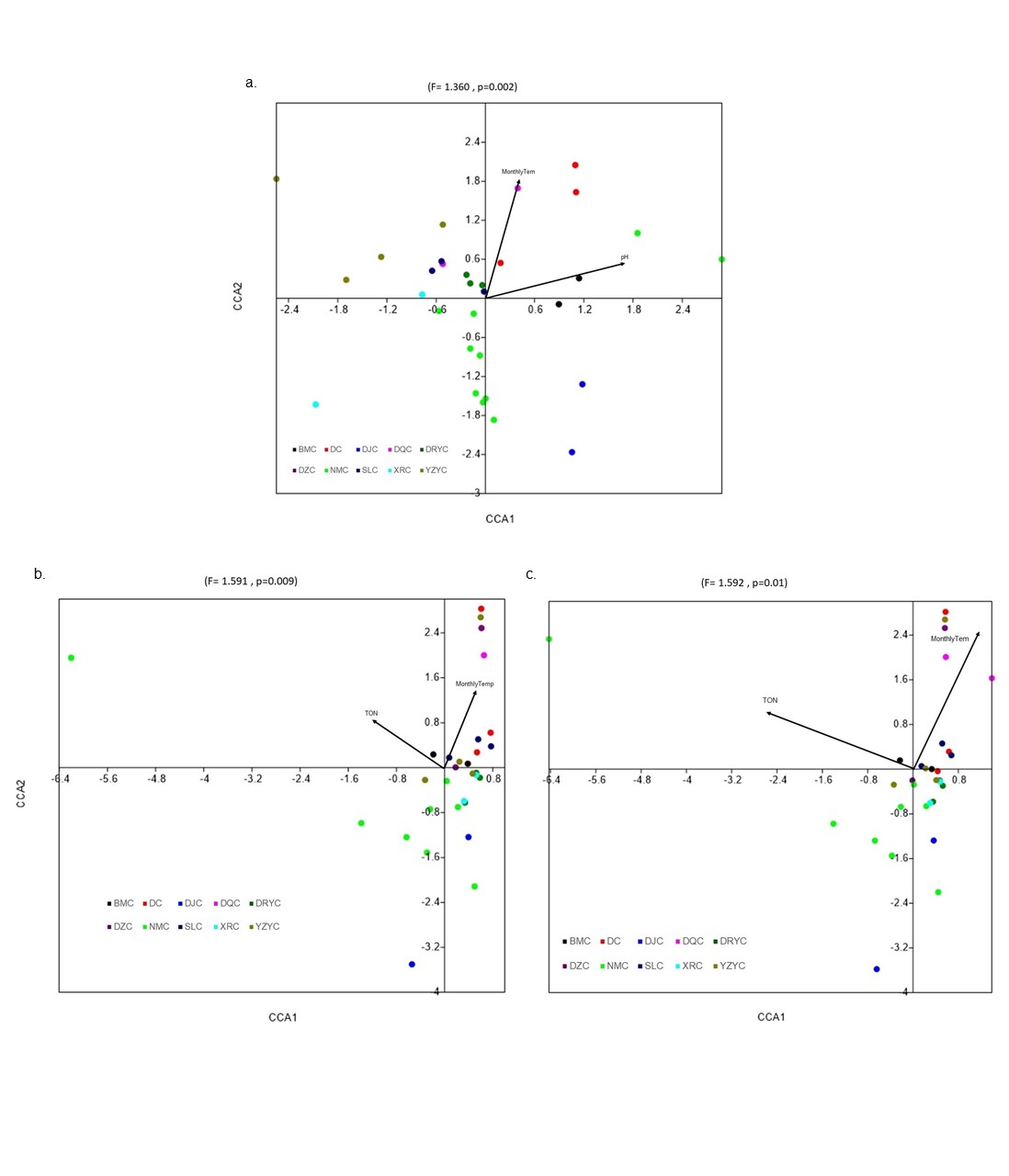


**Figure S3.** CCA analysis of microbial community (a), all SRP sub-community (b) and high abundant SRP only (c).

**Table S1**. The environmental factors and geographical features of the saline lakes.

|  | **Latitude** | **Longitude** |
| --- | --- | --- |
| BMC | 31°21′54.45″ to 31°21′55.65″N | 90°34′44.56″ to 90°37′54.95″E |
| DC | 31°35′17.05″ to 31°39′36.80″N | 91°05′12.65″ to 91°06′49.54″N |
| DJC | 29°46′27.96″ to 29°52′53.44″N | 85°44′57.78″ to 85°45′16.47″E |
| DQC | 31°31′57.35″ to 31°32′22.88″N | 86°43′43.02″ to 86°44′11.84″E |
| DRYC | 31°05′32.43″ to 31°21′40.66″N | 86°38′04.41″ to 86°42′17.63″E |
| DZC | 31°49′19.45″N | 87°33′06.49″E |
| NMC | 30°33′50.94″ to 30°55′36.01″N | 90°33′12.67″ to 91°02′46.58″E |
| SLC | 31°42′12.05″ to 31°48′23.23″N | 88°27′02.31″ to 89°21′55.96″E |
| XRC | 30°14′31.49″ to 30°18′33.64″N | 86°26′37.90″ to 86°28′22.70″N |
| YZYC | 28°58′29.53″ to 29°00′36.55″N | 90°55′11.63″ to 90°58′06.22″E |

**Table S2**. Primers for epicPCR and 16S rRNA gene PCR amplification.

| **Primers** | **Sequences** |
| --- | --- |
| **dsrB-F1** | GTGTAGCAGTTACCGCA |
| **R2 (1492R)** | GGTTACCTTGTTACGACTT |
| **dsrB-R1_519R** | GWATTACCGCGGCKGCTGTGCCTSAAYATGTGYGGYG |
| **dsrB-F3** | YRVAGV ATSGCGATRT CGGA |
| **E786R** | GGACTACH VGGGTWTCTA AT |
| **U519R-block10** | TTTTTTTTTTGWATTACCGCGGCKGCTG/3SpC3/ |
| **U519F-block10** | TTTTTTTTTTCAGCMGCCGCGGTAATWC/3SpC3/ |

**Table S3. barcodes for epicPCR and 16S rRNA amplicons**

| **Sample** | **epicPCR** | | **16S rRNA** | |
| --- | --- | --- | --- | --- |
|  | forward_barcode | reverse_barcode | forward_barcode | reverse_bacode |
| BMC1 | GTATTGGTCAGA | TTGGACGTCCAC | TCACCCAAGGTA | ACTCGGCCAACT |
| BMC2 | AGAACCGTCATA | TCCAGGGCTATA | AGCCAGTCATAC | GTTGGTTGGCAT |
| DC1 | TCACCCAAGGTA | CTACCGATTGCG | CAAACGCACTAA | GTAGTGTCAACA |
| DC2 | AGCCAGTCATAC | CCTACCATTGTT | GAACAAAGAGCG | TGGAGAGGAGAT |
| DC3 | AGCGAACCTGTT | GATAACTGTACG | GCTAAGTGATGT | CGTATAAATGCG |
| DJC1 | AGGGCTATAGTT | AAGATCGTACTG | AAGGGACAAGTG | AATACAGACCTG |
| DJC2 | TGTCTCGCAAGC | ACTCATCTTCCA | AGTGTCGATTCG | GACTCAACCAGT |
| DQC2 | GTTGATACGATG | CGCATACGACCT | CAGCCGCATATC | GAGATACAGTTC |
| DQC3 | CAGCCGCATATC | GAGATACAGTTC | CTATTAAGCGGC | GGAAGAAGTAGC |
| DRYC1 | CCAAGATTCGCC | GATCTAATCGAG | GAGTCCGTTGCT | ACACCGCACAAT |
| DRYC2 | GAGGCTGATTTA | AATCTTGCGCCG | GATAACTGTACG | GTCTCCTCCCTT |
| DRYC3 | TGTAGTATAGGC | GACCGTCAATAC | CCGAATTGACAA | CACCTGTAGTAG |
| DZC1 | CTCACGCAATGC | TTGGAACGGCTT | CTGGCATCTAGC | CACGAGCTACTC |
| DZC2 | GTCCCGTGAAAT | TCCTAGGTCCGA | ACTATGGGCTAA | TAGACACCGTGT |
| NMC1 | GGACAGTGTATT | TCCTCACTATCA | GCATTGAGTTCG | AGACAAGCTTCC |
| NMC2 | TGAACTAGCGTC | ATAAAGAGGAGG | CTATGGTGAACC | TCACTTGGTGCG |
| NMC3 | TCCTCTTTGGTC | ATCCCAGCATGC | GTATTGGTCAGA | TTGGACGTCCAC |
| NMC4 | GGCATTAGTTGA | TAAACGCGACTC | AGAACCGTCATA | TCCAGGGCTATA |
| NMC5 | CGGTAGTTGATC | CCTCGGGTACTA | ATACTCGGCTGC | GAAACTCCTAGA |
| NMC6 | TGAAAGCGGCGA | TTCACCTGTATC | AGCTTACCGACC | TACGCCCATCAG |
| NMC7 | GGTTACGGTTAC | CTCCAGGTCATG | AGGGCTATAGTT | AAGATCGTACTG |
| NMC8 | GTTGATACGATG | CAGGATTCGTAC | TGTCTCGCAAGC | ACTCATCTTCCA |
| SLC1 | CAGACACTTCCG | GCCTCGTACTGA | CCAAGATTCGCC | GATCTAATCGAG |
| SLC3 | CAGAAGGTGTGG | TTCCCTTCTCCG | TGTAGTATAGGC | GACCGTCAATAC |
| SLC4 | GAAGCTTGAATC | CATTTGACGACG | CTCACGCAATGC | TTGGAACGGCTT |
| XRC1 | GTTTGCTCGAGA | TAAACCTGGACA | GTCCCGTGAAAT | TCCTAGGTCCGA |
| XRC2 | CAAACGCACTAA | CCGAATTGACAA | GGACAGTGTATT | TCCTCACTATCA |
| YZYC1 | GAACAAAGAGCG | CTGGCATCTAGC | TGAACTAGCGTC | ATAAAGAGGAGG |
| YZYC2 | GCTAAGTGATGT | GGTGGTCGTTCT | TCCTCTTTGGTC | ATCCCAGCATGC |
| YZYC3 | AAGGGACAAGTG | ACTATGGGCTAA | GGCATTAGTTGA | TAAACGCGACTC |
| YZYC4 | AGTGTCGATTCG | GCATTGAGTTCG | CGGTAGTTGATC | CCTCGGGTACTA |

**Table S4 Accession number for Biosamples in NCBI**

| **Sample** | **16S rRNA gene sequence file** | **epicPCR sequence file** |
| --- | --- | --- |
| BMC1 | [SAMN09949769](https://www.ncbi.nlm.nih.gov/biosample/SAMN09949769) | [SAMN09949800](https://www.ncbi.nlm.nih.gov/biosample/SAMN09949800) |
| BMC2 | [SAMN09949770](https://www.ncbi.nlm.nih.gov/biosample/SAMN09949770) | [SAMN09949801](https://www.ncbi.nlm.nih.gov/biosample/SAMN09949801) |
| DC1 | [SAMN09949771](https://www.ncbi.nlm.nih.gov/biosample/SAMN09949771) | [SAMN09949802](https://www.ncbi.nlm.nih.gov/biosample/SAMN09949802) |
| DC2 | [SAMN09949772](https://www.ncbi.nlm.nih.gov/biosample/SAMN09949772) | [SAMN09949803](https://www.ncbi.nlm.nih.gov/biosample/SAMN09949803) |
| DC3 | [SAMN09949773](https://www.ncbi.nlm.nih.gov/biosample/SAMN09949773) | [SAMN09949804](https://www.ncbi.nlm.nih.gov/biosample/SAMN09949804) |
| DJC1 | [SAMN09949774](https://www.ncbi.nlm.nih.gov/biosample/SAMN09949774) | [SAMN09949805](https://www.ncbi.nlm.nih.gov/biosample/SAMN09949805) |
| DJC2 | [SAMN09949775](https://www.ncbi.nlm.nih.gov/biosample/SAMN09949775) | [SAMN09949806](https://www.ncbi.nlm.nih.gov/biosample/SAMN09949806) |
| DQC2 | [SAMN09949790](https://www.ncbi.nlm.nih.gov/biosample/SAMN09949790) | [SAMN09949807](https://www.ncbi.nlm.nih.gov/biosample/SAMN09949807) |
| DQC3 | [SAMN09949776](https://www.ncbi.nlm.nih.gov/biosample/SAMN09949776) | [SAMN09949808](https://www.ncbi.nlm.nih.gov/biosample/SAMN09949808) |
| DRYC1 | [SAMN09949777](https://www.ncbi.nlm.nih.gov/biosample/SAMN09949777) | [SAMN09949809](https://www.ncbi.nlm.nih.gov/biosample/SAMN09949809) |
| DRYC2 | [SAMN09949778](https://www.ncbi.nlm.nih.gov/biosample/SAMN09949778) | [SAMN09949810](https://www.ncbi.nlm.nih.gov/biosample/SAMN09949810) |
| DRYC3 | [SAMN09949779](https://www.ncbi.nlm.nih.gov/biosample/SAMN09949779) | [SAMN09949811](https://www.ncbi.nlm.nih.gov/biosample/SAMN09949811) |
| DZC1 | [SAMN09949780](https://www.ncbi.nlm.nih.gov/biosample/SAMN09949780) | [SAMN09949812](https://www.ncbi.nlm.nih.gov/biosample/SAMN09949812) |
| DZC2 | [SAMN09949781](https://www.ncbi.nlm.nih.gov/biosample/SAMN09949781) | [SAMN09949813](https://www.ncbi.nlm.nih.gov/biosample/SAMN09949813) |
| NMC1 | [SAMN09949782](https://www.ncbi.nlm.nih.gov/biosample/SAMN09949782) | [SAMN09949814](https://www.ncbi.nlm.nih.gov/biosample/SAMN09949814) |
| NMC2 | [SAMN09949783](https://www.ncbi.nlm.nih.gov/biosample/SAMN09949783) | [SAMN09949815](https://www.ncbi.nlm.nih.gov/biosample/SAMN09949815) |
| NMC3 | [SAMN09949784](https://www.ncbi.nlm.nih.gov/biosample/SAMN09949784) | [SAMN09949816](https://www.ncbi.nlm.nih.gov/biosample/SAMN09949816) |
| NMC4 | [SAMN09949785](https://www.ncbi.nlm.nih.gov/biosample/SAMN09949785) | [SAMN09949817](https://www.ncbi.nlm.nih.gov/biosample/SAMN09949817) |
| NMC5 | [SAMN09949786](https://www.ncbi.nlm.nih.gov/biosample/SAMN09949786) | [SAMN09949818](https://www.ncbi.nlm.nih.gov/biosample/SAMN09949818) |
| NMC6 | [SAMN09949787](https://www.ncbi.nlm.nih.gov/biosample/SAMN09949787) | [SAMN09949819](https://www.ncbi.nlm.nih.gov/biosample/SAMN09949819) |
| NMC7 | [SAMN09949788](https://www.ncbi.nlm.nih.gov/biosample/SAMN09949788) | [SAMN09949820](https://www.ncbi.nlm.nih.gov/biosample/SAMN09949820) |
| NMC8 | [SAMN09949789](https://www.ncbi.nlm.nih.gov/biosample/SAMN09949789) | [SAMN09949821](https://www.ncbi.nlm.nih.gov/biosample/SAMN09949821) |
| SLC1 | [SAMN09949791](https://www.ncbi.nlm.nih.gov/biosample/SAMN09949791) | [SAMN09949822](https://www.ncbi.nlm.nih.gov/biosample/SAMN09949822) |
| SLC3 | [SAMN09949792](https://www.ncbi.nlm.nih.gov/biosample/SAMN09949792) | [SAMN09949823](https://www.ncbi.nlm.nih.gov/biosample/SAMN09949823) |
| SLC4 | [SAMN09949793](https://www.ncbi.nlm.nih.gov/biosample/SAMN09949793) | [SAMN09949824](https://www.ncbi.nlm.nih.gov/biosample/SAMN09949824) |
| XRC1 | [SAMN09949794](https://www.ncbi.nlm.nih.gov/biosample/SAMN09949794) | [SAMN09949825](https://www.ncbi.nlm.nih.gov/biosample/SAMN09949825) |
| XRC2 | [SAMN09949795](https://www.ncbi.nlm.nih.gov/biosample/SAMN09949795) | [SAMN09949826](https://www.ncbi.nlm.nih.gov/biosample/SAMN09949826) |
| YZYC1 | [SAMN09949796](https://www.ncbi.nlm.nih.gov/biosample/SAMN09949796) | [SAMN09949827](https://www.ncbi.nlm.nih.gov/biosample/SAMN09949827) |
| YZYC2 | [SAMN09949797](https://www.ncbi.nlm.nih.gov/biosample/SAMN09949797) | SAMN09949828 |
| YZYC3 | [SAMN09949798](https://www.ncbi.nlm.nih.gov/biosample/SAMN09949798) | [SAMN09949829](https://www.ncbi.nlm.nih.gov/biosample/SAMN09949829) |
| YZYC4 | [SAMN09949799](https://www.ncbi.nlm.nih.gov/biosample/SAMN09949799) | [SAMN09949830](https://www.ncbi.nlm.nih.gov/biosample/SAMN09949830) |

**Table S5**. The environmental factors and geographical features of the saline lakes. For assessing values of pH, SO_4_^2-^, salinity, TN and TOC, three replicates were measured for each sample. Abbreviations: SO_4_^2-^, sulfate concentration; TN, Total nitrogen; TOC, total organic carbon; MMT(Aug), average temperature of August 2015.

|  | **pH** | **SO_4_^2-^ (g/L)** | **salinity (g/L)** | **TN (g/kg)** | **TOC (%)** | **MMT(Aug) (°C)** |
| --- | --- | --- | --- | --- | --- | --- |
| BMC | 9.35±0.01 | 1.24±0.14 | 3.90±0.35 | 0.48±0.13 | 0.6±0.03 | 12.58 |
| DC | 9.51±0.01 | 4.93±0.20 | 12.27±0.62 | 0.49±0.30 | 0.54±0.30 | 14.75 |
| DJC | 9.27±0.04 | 0.88±0.06 | 2.17±0.13 | 0.09±0.02 | 0.22±0.12 | 9.95 |
| DQC | 9.32±0.01 | 5.23±0.06 | 122.39±1.26 | 0.08±0.04 | 0.38±0.01 | 14.29 |
| DRYC | 9.15±0.01 | 2.17±0.77 | 5.75±1.98 | 0.09±0.05 | 0.14±0.05 | 12.88 |
| DZC | 9.68±0.01 | 5.25±0.11 | 13.76±0.80 | 0.07±0.03 | 0.11±0.02 | 13.75 |
| NMC | 9.04±0.04 | 0.15±0.04 | 0.44±0.14 | 0.87±0.80 | 0.81±1.21 | 10.90 |
| SLC | 9.12±0.04 | 1.65±1.56 | 3.81±1.02 | 0.53±0.49 | 0.62±0.64 | 13.13 |
| XRC | 8.90±0.04 | 0.45±0.01 | 1.99±0.03 | 0.21±0.16 | 0.25±0.11 | 10.43 |
| YZYC | 8.86±0.02 | 0.75±0.03 | 1.37±0.07 | 0.88±0.31 | 2.08±1.09 | 13.36 |

**Table S6.** The number of remained epicPCR reads for the quality checking processing.

| **Processing tools** | **Sequence type** | **Retained reads No.** | **Total reads No.** | **Removal Rate** |
| --- | --- | --- | --- | --- |
| **Detect barcode** | Total | 2702370 | 3693055 | 26.8% |
| **Trim primer** | R1 | 1393434 | 2702370 | 48.4% |
|  | R2 | 1413479 | 2702370 | 47.7% |
| **Flash** | R1 | 1318387 | 1393434 | 5.4% |
|  | R2 | 1318387 | 1413479 | 6.7% |
| **Trim bridging primer** | Total | 1138016 | 1318387 | 13.7% |
| **Trim N** | Total | 922926 | 1138016 | 18.9 |

**Table S7** The distribution of core and lake-specific OTUs shows endemism for microbial communities and SRPs across lakes.

|  | **Microbial community** | **SRP sub-community** | **high abundant SRP**  (>1% in at least one sample) |
| --- | --- | --- | --- |
| **OTUs** | 12,519 | 883 | 120 |
| **Core OTUs** | 74 | 18 | 2 |
| **Lake-specific OTUs** | 8463 | 615 | 91 |

**Table S8**. F and P values of CCA analysis. The *P* values of statistical significance (<0.05) are in bold.

|  | **Microbial community** | | **SRP sub-community** | | **high abundant SRP populations** | |
| --- | --- | --- | --- | --- | --- | --- |
|  | *F* | *P* | *F* | *P* | *F* | *P* |
| **pH** | 1.495 | **0.002** | 0.644 | 0.96 | 0.599 | 0.951 |
| **MMT(Aug)** | 1.15 | 0.116 | 1.293 | 0.084 | 1.3 | 0.097 |
| **TN** | 0.882 | 0.68 | 1.899 | **0.03** | 1.98 | **0.018** |
| **TOC** | 1.215 | 0.169 | 0.738 | 0.746 | 0.797 | 0.672 |
| **SO_4_^2-^** | 1.226 | 0.061 | 1.127 | 0.28 | 1.028 | 0.418 |
| **Salinity** | 1.052 | 0.386 | 1.279 | 0.228 | 0.616 | 0.615 |
